# Supplementary material for: Full-length direct RNA sequencing reveals extensive remodeling of RNA expression, processing and modification in aging Caenorhabditis elegans
Source: bioRxiv. 2024 Jun 22:2024.06.18.599640. Preprint. [Version 1] doi: 10.1101/2024.06.18.599640 (PMC11213008; doi:10.1101/2024.06.18.599640)
Supplement: Supplement 1 — Document S1. Figures S1–S7 Table S1. Illumina RNA-seq DESeq2 results from days 2, 3, 4, 5, 7, 10, and 15 vs. day 1. Table S2. Nanopore DRS DESeq2 results using unfiltered reads from days 2, 3, 4, 5, 7, 10, and 15 vs. day 1. Table S3. Number of Nanopore DRS reads per replicate remaining after each filtering step (mapping, transcription start site filtering, and poly(A) filtering). Table S4. All detected Nanopore DRS full-length isoforms with columns 1-12 in BED format and the number of filtered reads for each isoform per replicate. Novel isoforms not in the WS279 WormBase annotation are labeled as productive if they span in frame annotated start and stop codons or unproductive if they do not span in frame annotated start and stop codons. Table S5. 3’UTR coordinates of Nanopore DRS full-length isoforms with columns 1-12 in BED format. 3’UTR sequences are compared to the WS279 WormBase annotation. Table S6. Alternative exon inclusion events with a significant (P<0.05) change in relative abundance (PSI) in days 2, 3, 4, 5, 7, 10, or 15 compared to day 1. Table S7. Percent of reads mapping to introns or intergenic regions and percent of splice junction reads that do not match WS279 WormBase annotations in young (days 1, 2, 3) and old (days 7, 10, 15) animals. Table S8. Median, mean, minimum, and maximum poly(A) tail lengths for isoforms with full-length support. Table S9. High confidence A-to-G edit site coordinates in BED format, features that overlap with A-to-G sites, and predicted codon changes resulting from editing. Table S10. High confidence pseudouridine edit site coordinates in BED format and overlapping features. Table S11. Annotated aging genes with novel features from this study indicated. [file media-1.zip › supplementary_files/Schiksnis_Cell_Reports_Document_S1.pdf]

Supplemental Information

Full-length direct RNA sequencing reveals extensive remodeling of RNA expression, processing and modification in aging *Caenorhabditis elegans*

Erin C. Schiksnis, Ian A. Nicastro, and Amy E. Pasquinelli

Supplementary Figures

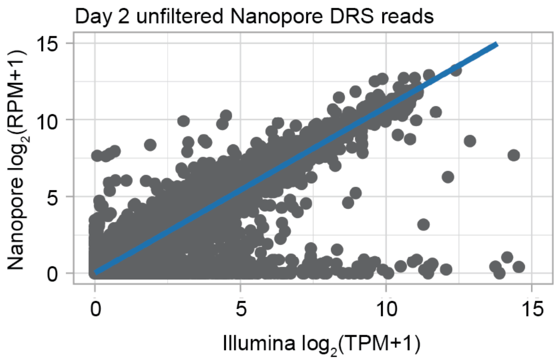

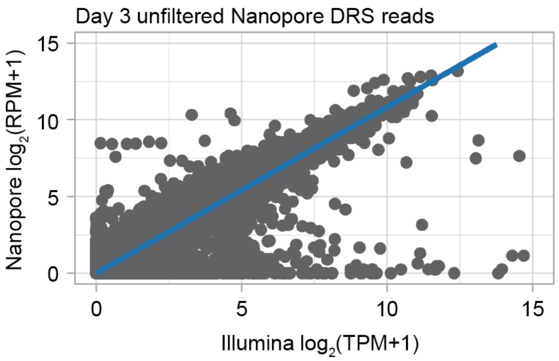

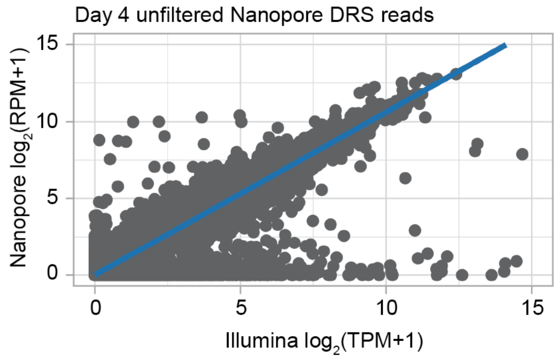

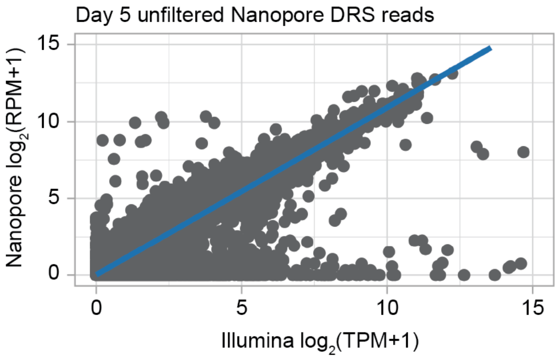

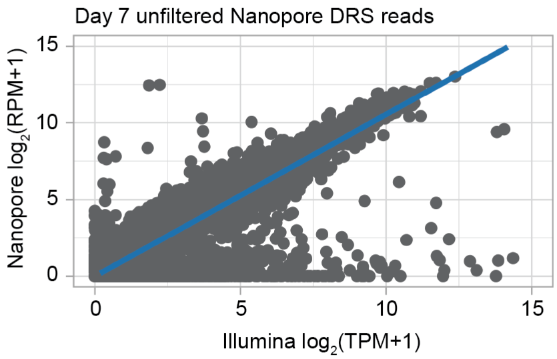

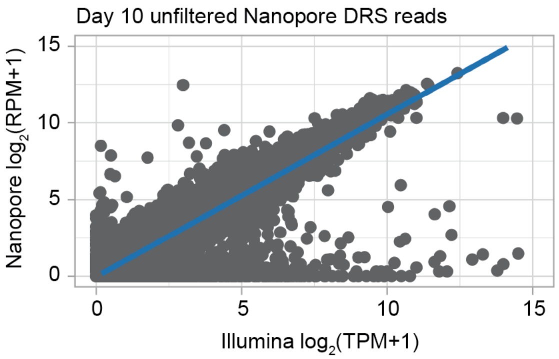

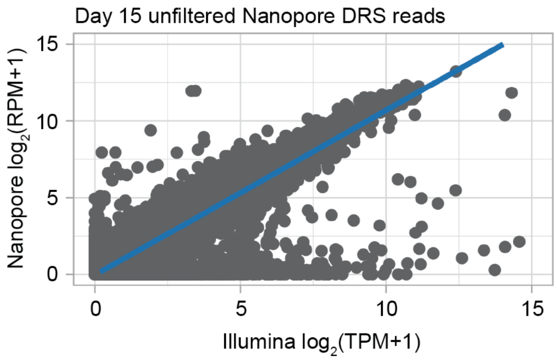

Figure S1, related to Figure 1. Correlations between Nanopore DRS and Illumina RNA-seq normalized gene expression. Scatterplots comparing mean normalized expression of individual genes across three independent biological replicates between Nanopore DRS (log_2 TPM + 1) and Illumina RNA-seq (log_2 RPM + 1) at each time point.

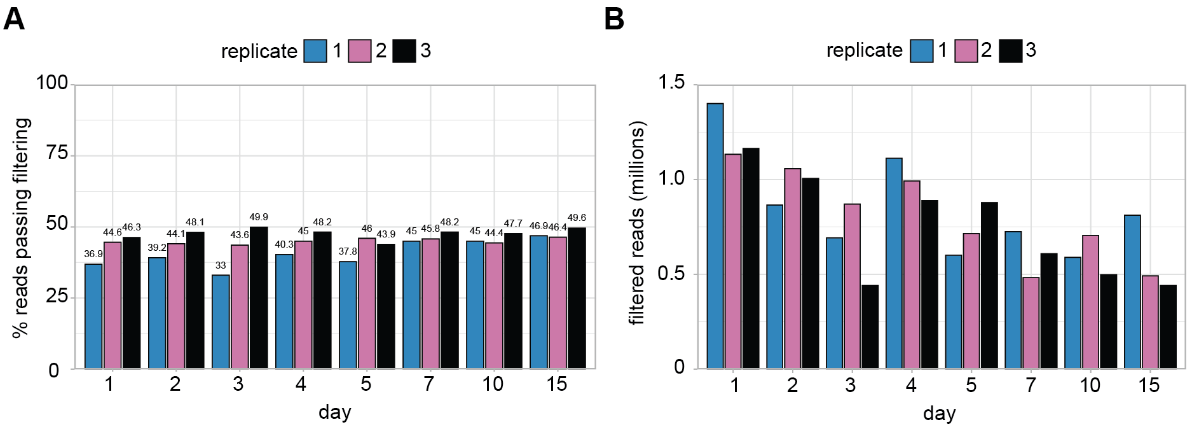

C

Day	Nanopore (full-length only) and Illumina correlation (R^2)
1	0.51
2	0.53
3	0.52
4	0.54
5	0.54
7	0.50
10	0.48
15	0.48

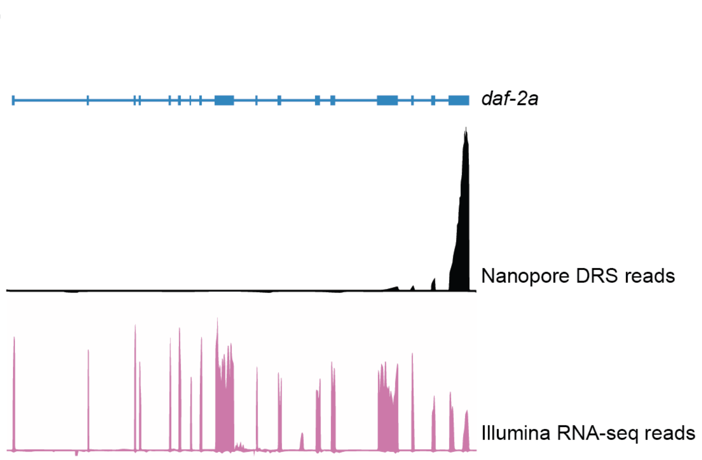

Figure S2, related to Figure 2. Read filtering for Nanopore DRS. A. Percent of reads that remain after Nanopore filtering pipeline at each time point. **B.** Number of reads that remain after Nanopore filtering pipeline at each time point. **C.** Table showing the R-squared value for mean normalized expression for filtered Nanopore DRS (log_2 TPM + 1) and Illumina RNA-seq (log_2 RPM + 1) of individual genes across three independent biological replicates at each time point. **D.** Browser track image of *daf-2a* showing unfiltered Nanopore DRS and Illumina RNA-seq read density.

A

Gene fusion	Total counts	Break 1	Break 2
C18H9.6:*clec-173*	728	II:6701465 (-)	IV:3266420 (-)
T28F3.8:*clec-84*	293	IV:17307321 (-)	IV:2845291 (+)
Y51H4A.24:*clec-84*	62	IV:16739384 (-)	IV:2845291 (+)
eri-6:*eri-7*	57	I:4458645 (+)	I:4456339 (-)
clec-84:K08D10.14	66	IV:2845291 (+)	I:4456339 (-)

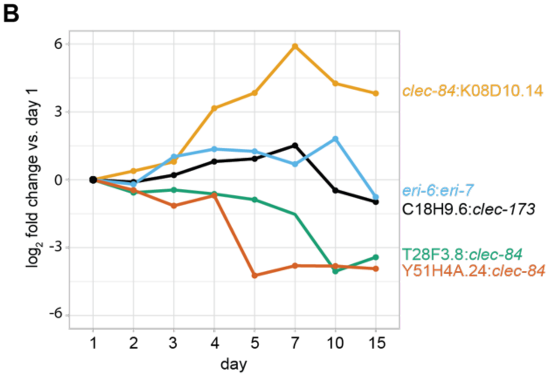

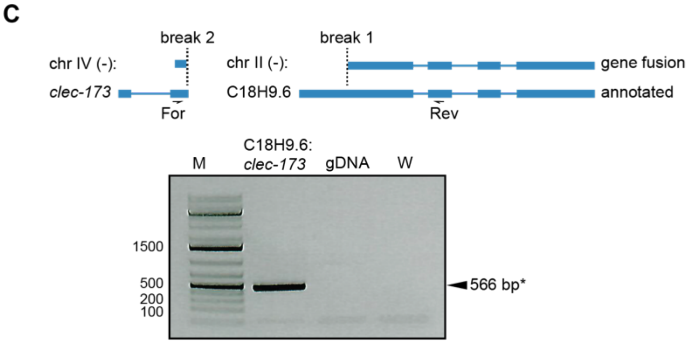

Figure S3, related to Figure 2. Detection of gene fusion isoforms at adult time points. A. Table of gene fusions detected with LongGF. Break points indicate the genomic coordinates where two genes are joined. **B.** Line plot showing the relative expression normalized to day 1 of LongGF fusion genes. **C.** RT-PCR validation of the C18H9.6:*clec-173* LongGF fusion isoform. Agarose gel electrophoresis (*bottom*) with expected fragment size denoted, including genomic DNA and water controls. Schematic representations of gene fusion (*top*) showing break points and forward and reverse PCR primers used for amplification of cDNA.

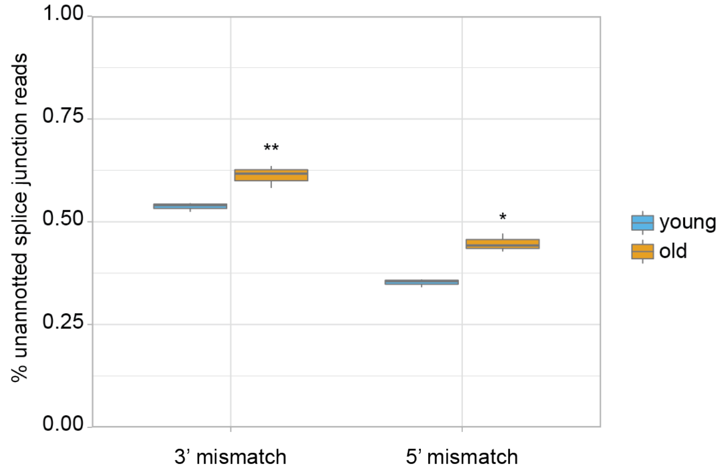

Figure S4, related to Figure 4. Percent of splice junction reads using unannotated 5’ or 3’ splice sites. Boxplot showing the percent of splice junction reads that do not match WS279 annotated 5’ splice sites (***P*=0.004, *t*-test, two-sided after probit transformation) or 3’ splice sites (**P*=0.022, *t*-test, two-sided after probit transformation).

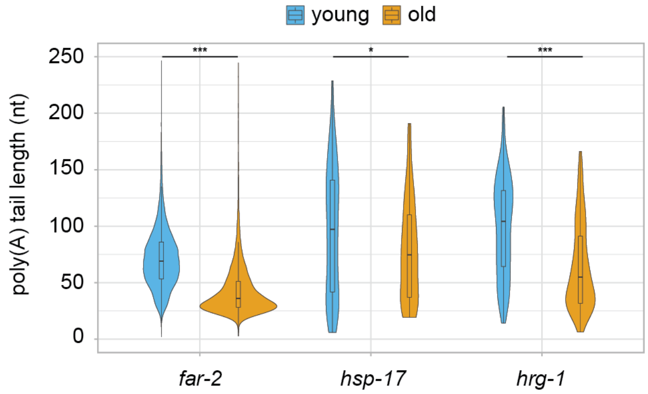

	far-2	hsp-17	hrg-1
**Δ median poly(A) (nt):**	-32	-21	-49
**log_2 fold change (old vs. young):**	-0.37	-0.04	0.01

Figure S5, related to Figure 4. Genes with large changes to poly(A) tail lengths in aging. Violin plot with inlaid boxplot of poly(A) tail length distribution for reads assigned to indicated genes. Changes to media poly(A) tail lengths and expression in old compared to young animals are indicated. ****P*<0.001, **P*<0.05 (Kolmogorov-Smirnov test) between young and old animals.

A

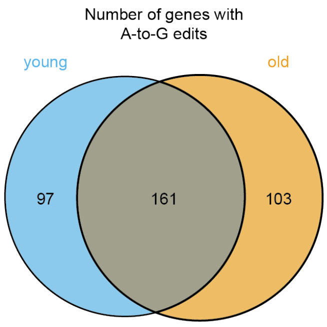

B

Amino acid group	Amino acids
Positively charged	R,H,K
Negatively charged	D,E
Polar	S,T,N,Q
Hydrophobic	A,V,I,L,M,F,Y,W
Special	C,G,P

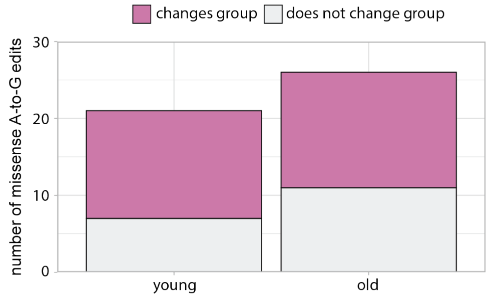

Figure S6, related to Figure 5. Examination of A-to-I editing in aging. A. Venn diagram with the number of genes with A-to-G edits in young and old animals. **B.** Table with amino acid group characterizations based on chemical properties (*left*) and stacked bar plot showing the number of predicted missense A-to-G edits that result in a change to the encoded amino acid group (*right*).

A

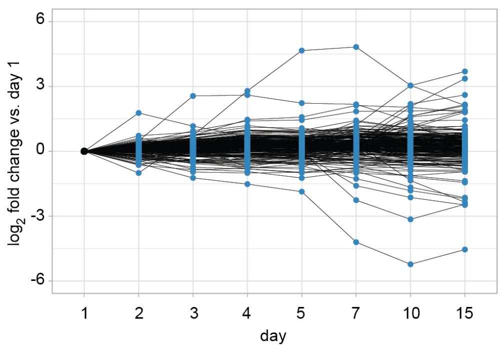

B

Gene	Human homolog	Target(s) of human homolog
pus-1	Pus1	tRNA
tag-124	Pus3	tRNA, mRNA
Y43B11AR.3	TruB2	tRNA, mt-mRNA
K07E8.7	RpusD2	mRNA
ZK287.7	RpusD3, RPusD4	mt-rRNA, tRNA
B0024.11	Pus7	tRNA, mRNA
Y48C3A.20	Pus10	tRNA, rRNA, snRNA

C

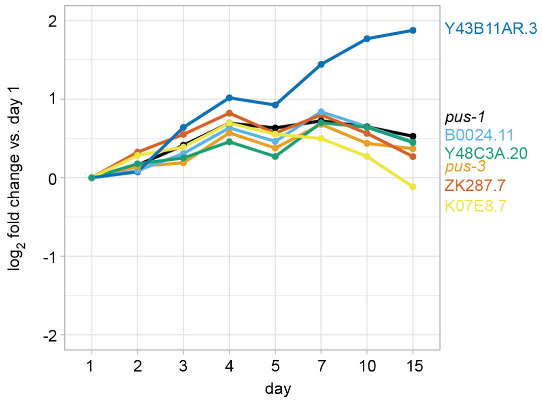

Figure S7, related to Figure 6. Dynamic expression of genes with pseudouridine and pseudouridine synthase enzymes. A. Line plot showing the relative expression normalized to day 1 of genes with pseudouridine in old animals but not young. Each blue dot represents an individual gene. **B.** Table of all predicted *C. elegans* homologs of human pseudouridine synthase enzymes and their RNA targets in human. **C.** Line plot showing the relative expression normalized to day 1 of predicted pseudouridine synthase genes.
